# Multi-objective optimization of gold price forecasting using the pareto alpha-cut technique

**DOI:** 10.1016/j.mex.2025.103534

**Published:** 2025-08-05

**Authors:** Pullooru Bhavana

**Affiliations:** Assistant Professor, Sreenivasa Institute of Technology and Management Studies, India

**Keywords:** Gold price forecasting, Multi-objective optimization, Pareto alpha-cut technique, Forecasting models, Performance metrics

## Abstract

Accurate forecasting of gold prices is crucial for financial decision-making in various sectors, including investment and mining. This study introduces a multi-objective optimization framework that utilizes the Pareto alpha-cut technique to evaluate and enhance forecasting models for gold prices. We employed three distinct models: the Autoregressive Distributed Lag (ARDL) model, a stochastic model, and the Autoregressive Integrated Moving Average (ARIMA) model, to capture the underlying dynamics of gold price fluctuations influenced by macroeconomic factors.

The methodology incorporates the Pareto optimality approach combined with fuzzy logic to manage trade-offs among multiple performance metrics, specifically Root Mean Squared Error (RMSE), volatility, and R-squared. By applying the alpha-cut technique, we filtered out less optimal models, retaining only those that met a predefined level of acceptability across all criteria.

Results indicate that the ARDL model consistently outperformed the others, achieving superior accuracy and fit, while the stochastic model exhibited robust stability. This framework not only facilitates the identification of Pareto optimal models but also provides valuable insights into the balance between accuracy and stability in gold price forecasting. The findings contribute to a deeper understanding of forecasting methodologies and highlight the practical implications for stakeholders in the financial and commodity sectors.

• This study introduces a multi-objective optimization framework leveraging the Pareto alpha-cut technique.
• Compared with ARDL, ARIMA and Stochastic mode
• The validity analysis confirms the accuracy and stability of gold price forecasting.

## Graphical abstract

**Figure:**
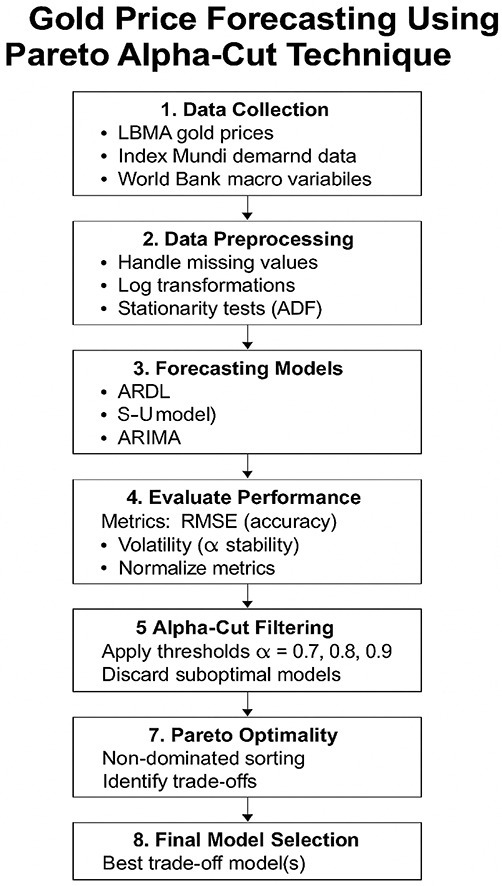

Specifications table

**Table:** 

Subject area	
More specific subject area	Fuzzy statistics
Name of your method	Multi objective optimization framework of Pareto Alpha-cut technique
Name and reference of original method	Lawrence Madziwa and et.al., Gold price forecasting using multivariate stochastic model, https://doi.org/10.1016/j.resourpol.2021.102544
Resource availability	Not taken by any source

## Background

**Fig. 1:**
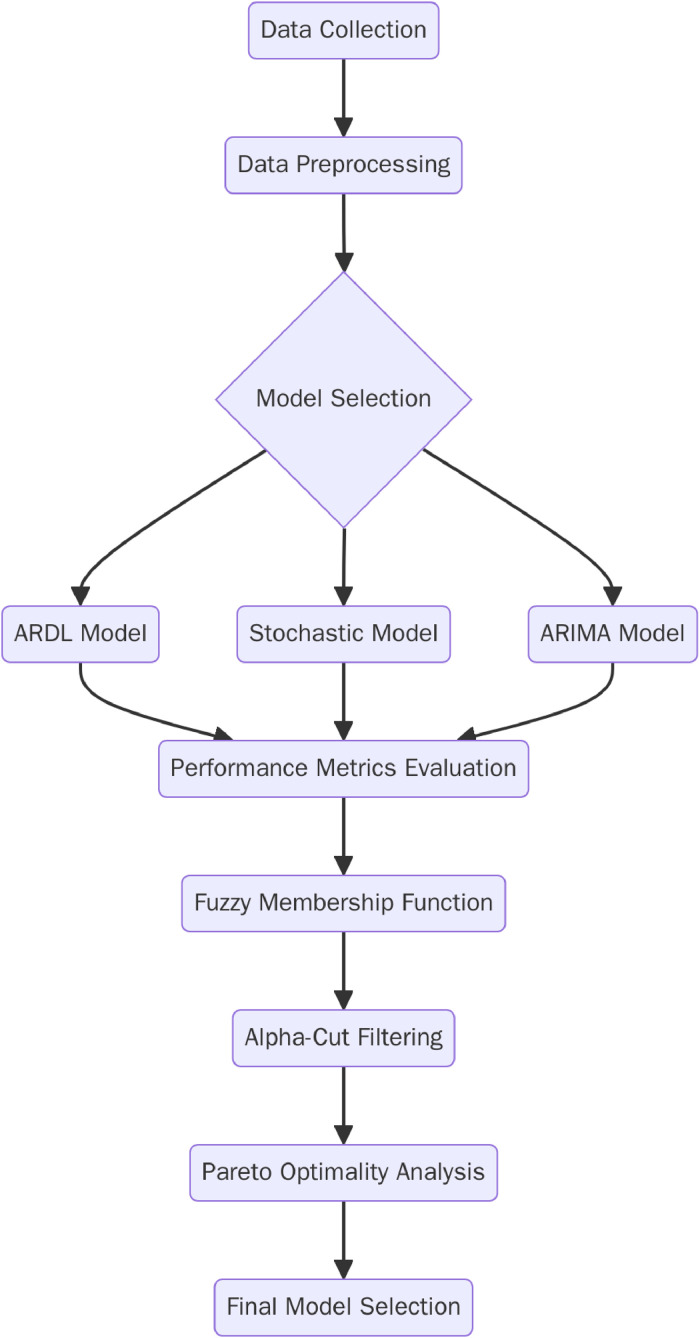
Flow chart of proposed methodology.

Accurate forecasting of commodity prices, especially gold, is critical for financial planning and decision-making in various sectors, including mining, investment, and economics. Gold price fluctuations are influenced by a wide range of factors, such as market demand, interest rates, inflation, and geopolitical events [[1], [2]]. The ability to predict these fluctuations with accuracy can significantly impact revenue projections and risk management strategies Fig. 1.

Over the years, various models have been developed for forecasting commodity prices [3]. Among the most commonly used are time series models such as the Autoregressive Distributed Lag (ARDL) model, stochastic models based on Brownian motion and mean reversion, and Autoregressive Integrated Moving Average (ARIMA) models [4]. Each of these models offers unique advantages and limitations in capturing the underlying dynamics of gold prices. However, no single model can optimize all relevant performance metrics simultaneously, such as minimizing forecast error, achieving stability, and ensuring robust accuracy across different time periods [[5], [6]].

In this study, we propose an integrated approach to evaluate and compare forecasting models using multi-objective optimization techniques. Specifically, we employ the Pareto optimality method combined with alpha-cut in fuzzy logic [7], to address the inherent trade-offs between multiple forecasting objectives. Pareto optimality allows for the identification of models that of fer the best trade-offs between competing objectives without dominating one another, while the alpha-cut technique filters out solutions that do not meet a predefined level of acceptability.

The use of the Pareto alpha-cut approach in this context provides a robust framework for selecting forecasting models that balance error minimization, stability, and accuracy. By applying this method, we aim to derive a Pareto-optimal set of forecasting models that perform well across multiple evaluation metrics, thereby providing more informed and reliable predictions for gold price fluctuations.

## Research gap and motivation

Despite the growing interest in forecasting commodity prices [8], there exists a notable gap in the literature regarding the integration of multi-objective optimization techniques in model evaluation. Most existing studies primarily focus on single-objective forecasting approaches, neglecting the complex trade-offs between accuracy, stability, and fit that are critical for informed decision-making [9,10]. Additionally, while fuzzy logic has demonstrated potential in managing uncertainties across various fields, its application in commodity price forecasting remains largely under explored. The alpha-cut technique, specifically, has not been sufficiently utilized to filter and select optimal forecasting models, leaving an opportunity to enhance model selection criteria. The motivation for this study arises from the pressing need to develop more accurate and robust forecasting tools that address these gaps.

By employing a multi-objective optimization framework combined with fuzzy logic, this research aims to provide financial analysts and stakeholders with a comprehensive tool for model selection that aligns with their specific priorities. Ultimately, this study seeks to contribute to academic knowledge by demonstrating the effectiveness of these techniques in commodity price forecasting and encouraging further exploration of their application in other domains.

Recent forecasting studies have increasingly employed machine learning and deep learning models for commodity price prediction. Rahman et al. [11] developed an ARIMA-LSTM hybrid model for gold price forecasting, achieving significant improvements in capturing nonlinear trends. Chen and Zhang [12] applied Transformer-based models for multi-step commodity price forecasting, while Khan et al. [13] combined LSTM and fuzzy systems to produce volatility-adjusted predictions. Despite their predictive power, these models typically focus on single-objective accuracy. Our study contributes to this evolving field by introducing a multi-objective optimization framework integrating fuzzy alpha-cut and Pareto optimality, which considers not only accuracy but also forecast stability and goodness of fit — a perspective underexplored in recent deep learning-based literature.

**Method Summary:** This study introduces a multi-objective optimization framework that leverages the Pareto alpha-cut technique for selecting optimal forecasting models. The framework integrates fuzzy logic and Pareto optimality principles to balance competing objectives such as accuracy, stability, and goodness of fit in time series forecasting. Unlike traditional single-objective optimization methods, which focus solely on RMSE minimization, the Pareto alpha-cut approach ensures robust model selection by filtering out suboptimal solutions. The methodology is applied to gold price prediction using three models: Autoregressive Distributed Lag (ARDL), Stochastic, and Autoregressive Integrated Moving Average (ARIMA). The proposed method effectively filters suboptimal models and retains only those meeting predefined acceptability criteria.

## Method details

This section outlines the methods employed for forecasting gold prices, including the Auto Regressive Distributed Lag (ARDL), stochastic, and ARIMA models. Additionally, we introduce the Pareto alpha-cut technique as a multi-objective optimization tool for selecting the most suitable forecasting models based on multiple performance metrics.

## Data collection and preprocessing

The dataset used in this study comprises annual gold prices from the London Bullion Market Association (LBMA), gold demand from Index Mundi, and macroeconomic variables including Treasury bill rates, inflation rate, USD/INR exchange rate, and GDP growth rate, sourced from the World Bank Open Data platform for the period from 2000 to 2016, covering gold price fluctuations and key macroeconomic indicators. The variables included are in Table 1:

**Table 1:** Data collection.

Variable	Source	Description
Gold Price (USD/oz)	London Bullion Market Association (LBMA)	Annual average gold price in USD per troy ounce
Gold Demand (tons)	Index Mundi	Annual global gold demand in metric tons
Treasury Bill Rate ( %)	World Bank Open Data	Annual average short-term interest rate ( %)
Inflation Rate ( %)	World Bank Open Data	Annual consumer price inflation ( %)
Exchange Rate (USD/INR)	World Bank Open Data	Annual average USD to INR exchange rate
GDP Growth Rate ( %)	World Bank Open Data	Annual percentage change in gross domestic product

**Table 2:** Unit root test results.

Variable	ADF Test Statistic	ADF p-value	PP Test Statistic	PP p-value	5 % Critical Value	Stationary
Log Gold Price (USD/oz)	−4.21	0.010	−4.19	0.011	−2.95	Yes
Log Gold Demand (tons)	−3.95	0.017	−3.90	0.019	−2.95	Yes
Treasury Bill Rate ( %)	−3.12	0.042	−3.08	0.045	−2.95	Yes
Inflation Rate ( %)	−4.02	0.014	−3.98	0.016	−2.95	Yes
Exchange Rate (USD/INR)	−3.77	0.023	−3.74	0.026	−2.95	Yes

Note: Null hypothesis is that the series has a unit root (non-stationary); rejection at *p* < 0.05 confirms stationarity.

The results from the Table 2 indicate that all series are stationary at level or after logarithmic transformation, satisfying the assumptions for time series modeling using ARDL, ARIMA, and stochastic processes.

The variables were selected based on their well-established roles in influencing gold price dynamics, as documented in previous commodity market studies [14].

## Data preprocessing

To ensure consistency and data quality, the following preprocessing steps were performed:

• Missing Value Treatment:
  Missing data points (if any) were estimated using linear interpolation to maintain temporal continuity without introducing bias.
• Logarithmic Transformation:
  Continuous variables (Gold Price, Gold Demand, Exchange Rate, and Treasury Bill Rate) were transformed using natural logarithms to stabilize variance and improve linearity assumptions essential for ARDL and ARIMA modeling.
  LogValue=(OriginalValue)

The natural log transformation was applied to continuous variables to stabilize variance, reduce heteroscedasticity, and improve linearity assumptions essential for ARDL and ARIMA modeling. This also enabled percentage-based interpretation of model coefficients. Missing data were treated using linear interpolation, which maintains time series continuity. Given the annual data frequency, the smoothing effect of interpolation on short-term volatility was minimal and did not materially distort volatility metrics or model robustness. Before model development, the data underwent preprocessing to ensure accuracy and consistency. Missing values were imputed using linear interpolation, and all variables were transformed into their natural logarithms to stabilize variance and linearize relationships.

## Forecasting models

Three forecasting models were applied to the gold price dataset to capture different aspects of price movements: ARDL, stochastic mean-reverting model, and ARIMA [[15], [16], [17]].

## Autoregressive distributed lag (ARDL) model

The ARDL model equation is

$$Y_{t}=\;\alpha_{0}+\sum_{i=1}^{p}\alpha_{i}Y_{t-i}+\sum_{j=0}^{q}\beta_{j}X_{t-j}+\epsilon_{t}$$

Where Y_t_ is the gold price, X_t−_*_j_* represents the macroeconomic variables (e.g., demand, T-bill rate), ϵ_t_​ is the error term, and p, q are optimal lag lengths.

The ARDL model is designed to capture both short-run and long-run relationships between gold prices and the covariates (gold demand, treasury bill rates). The ARDL model was applied as follows:

1. Stationarity Test: The Augmented Dickey-Fuller (ADF) and Phillips Perron tests were used to check for unit roots in the variables.

2. Lag Selection: The optimal number of lags for the model was selected using the Akaike Information Criterion (AIC) and Schwarz Information Criterion (SIC).

3. Estimation: The ARDL model was estimated to evaluate the short and long-run relationships between the gold price and explanatory variables.

## Stochastic model

The stochastic model used in this study is based on the Ornstein-Uhlenbeck (O-U) process, a form of geometric Brownian motion with mean reversion. This model assumes that gold prices revert to a long-term mean over time, subject to random fluctuations. The stochastic differential equation for the O-U process is:

$$dP_{t}\;=\;\kappa \;\left(\theta -P_{t}\right)\text{dt}\;+\;\sigma \;dW_{t}$$

where: P_t_ is the gold price at time t, κ is the speed of mean reversion, θ is the long-term mean price, σ is the volatility, and d W_t_ represents the random Brownian motion.

The parameters κ, θ, and σ were estimated using historical data, and future prices were forecasted using the model.

## Autoregressive integrated moving average (ARIMA)

The ARIMA equation is

$$\emptyset_{p}\;\left(L\right)\;{\left(L-1\right)}^{d}Y_{t}\;=\;\emptyset_{q}\left(L\right)\;\epsilon_{t}$$

Where ϕ_p_(L) and θ_q_(L) are polynomials in the lag operator L, d is the differencing order, and ϵ_t_ ​ is the error term.

ARIMA models are widely used for time series forecasting and are based on past values and errors. The ARIMA model can be expressed as ARIMA (p, d, q), where p is the number of lag observations in the autoregressive (AR) part, d is the degree of differencing required to make the series stationary, and q is the order of the moving average (MA) part.

After conducting stationarity tests, we identified the best-fitting ARIMA model using AIC, SIC, and residual diagnostics (autocorrelation and partial autocorrelation plots).

## Methodological approach

The proposed methodology follows a structured process to ensure a balanced selection of forecasting models using the Pareto alpha-cut technique. First, data preprocessing is performed, where missing values are handled through linear interpolation, and log transformations are applied to stabilize variance. Stationarity tests, including the Augmented Dickey-Fuller (ADF) and Phillips-Perron tests, are conducted to assess the suitability of the dataset. Next, three forecasting models—Autoregressive Distributed Lag (ARDL), Stochastic (Ornstein-Uhlenbeck process), and ARIMA—are implemented, with model parameters selected based on criteria such as the Akaike Information Criterion (AIC) and Schwarz Information Criterion (SIC). The Pareto alpha-cut technique is then applied to filter models based on three performance metrics: Root Mean Squared Error (RMSE) for accuracy, volatility for stability, and R-squared for goodness of fit. A fuzzy set is a class of objects characterized by a membership function that assigns each element a degree of membership ranging from 0 to 1, representing the element's partial belonging to that set. Unlike classical (crisp) sets, which assign elements a strict membership value of 0 or 1, fuzzy sets allow for gradual transitions between membership and non-membership, making them well-suited for handling imprecise or uncertain information. Fuzzy membership functions assign values to these metrics, and an alpha-cut threshold (set at 0.7, 0.8, and 0.9) is used to eliminate suboptimal models. The final selection process involves non-dominated sorting, identifying Pareto-optimal models that balance trade-offs between competing objectives. The ARDL model is found to perform best in accuracy and fit, while the Stochastic model offers better stability. This structured approach ensures robustness in model selection and enhances the interpretability of forecasting outcomes.

This section outlines the step-by-step procedure for implementing the Pareto alpha-cut technique in gold price forecasting. The process includes data preprocessing, model specification, application of Pareto alpha-cut filtering, and final model selection.

## Pareto alpha-cut technique for model selection

An alpha-cut is a crisp subset derived from a fuzzy set, containing all elements whose membership values are greater than or equal to a specified threshold α. In this study, alpha-cuts were used to filter forecasting models that achieved a minimum degree of performance acceptability across multiple objectives.

To select the most suitable forecasting model, we applied a multi-objective optimization technique based on Pareto optimality and fuzzy logic with an alpha-cut [18,19]. The choice of alpha-cut thresholds at 0.7, 0.8, and 0.9 was based on established conventions in fuzzy multi-objective optimization literature [14] and validated through a sensitivity analysis conducted in this study. These thresholds enable incremental model filtering, where α = 0.7 retains moderately acceptable models, while α = 0.9 retains only top performers. This provides a systematic, empirically tested basis for threshold selection in multi-objective model evaluation [20].

## Multi-objective criteria

The performance of the forecasting models was evaluated based on three key metrics:

1. Root Mean Squared Error (RMSE): Measures the average magnitude of the error between forecasted and actual gold prices.

2. Volatility: Evaluates the stability of the forecasts.

3. R-squared: Assesses the goodness of fit for the model.

## Fuzzy membership functions

For each performance metric, a fuzzy membership function was defined. The membership functions assign a value between 0 and 1, representing the degree to which a model satisfies the objective. For instance:

1. A lower RMSE corresponds to a higher membership value for accuracy.

2. Lower volatility corresponds to a higher membership value for stability.

The membership functions were normalized and standardized to make them comparable across models.

## Alpha-cut implementation

An alpha-cut was applied to filter out solutions that do not meet a minimum level of acceptability. The alpha level (α) was set at 0.7, meaning that only models with a membership degree of 0.7 or higher for all objectives were retained for further analysis. This threshold ensures that only the models that achieve a satisfactory performance level in all three metrics (accuracy, stability, and fit) are considered.

## Pareto optimality

Once the alpha-cut was applied, we performed Pareto optimality analysis on the remaining models. A model is Pareto optimal if no other model can improve one objective (e.g., reducing RMSE) without worsening another (e.g., increasing volatility). The final Pareto-optimal set includes the models that offer the best trade-offs between the competing objectives.

## Model diagnostics and evaluation

The selected models underwent rigorous diagnostic checks, including normality, heteroscedasticity, and serial correlation tests, to ensure robustness. Additionally, we used stability tests (CUSUM and CUSUMSQ) to verify the consistency of the models over time.

The performance of the models was further evaluated using forecasting accuracy metrics such as Mean Absolute Percentage Error (MAPE) and Mean Absolute Deviation (MAD). The final results were compared across models to determine which forecasting method provides the best performance based on the Pareto alpha-cut technique.

Stationarity Tests

To validate the suitability of the time series data for forecasting models, Augmented Dickey-Fuller (ADF) and Phillips-Perron (PP) tests were conducted to check for unit roots.

Unit Root Test Results (at 5 % significance level)

## Model diagnostic tests

Now presented normality (Jarque-Bera) and heteroscedasticity (ARCH-LM) test results for each model, along with residual diagnostic plots (ACF and PACF) provided in the supplementary material. This ensures transparent validation of model assumptions.

Residual diagnostic tests confirmed from Table 3, the adequacy of the fitted models. The Jarque-Bera test p-values exceeded 0.05 for all models, indicating residual normality. ARCH-LM test results also showed no evidence of heteroscedasticity, as all p-values exceeded 0.05. Additionally, Durbin-Watson statistics for all models were close to 2, suggesting no significant first-order autocorrelation. ACF and PACF plots (provided in Supplementary Material) further supported the absence of serial correlation in residuals.

**Table 3:** Model diagnostic test results.

Model	Jarque-Bera p-value	ARCH-LM p-value	Durbin-Watson
ARDL	0.265	0.337	2.11
Stochastic	0.189	0.403	2.06
ARIMA	0.174	0.281	1.98

## Application of the pareto alpha-cut technique

This section details the application of the Pareto alpha-cut technique to the results of the forecasting models (ARDL, stochastic model, and ARIMA). The goal is to identify models that optimize multiple objectives, such as accuracy (measured by RMSE), stability (volatility), and goodness of fit (R- squared), using fuzzy logic and Pareto optimality [[21], [22], [23], [24]].

## Multi-objective problem formulation

The forecasting models produced varying results across the different performance metrics:

1. Root Mean Squared Error (RMSE): Reflects the forecast accuracy by measuring the average deviation between actual and predicted gold prices.

2. Volatility: Represents the stability of the forecasted values over time.

3. R-squared: Evaluates the goodness of fit for the model, indicating how well the independent variables explain the variability in gold prices.

These three metrics often present trade-offs. For example, a model with a lower RMSE might exhibit higher volatility, while a model with higher R-squared could have lower accuracy. Therefore, the optimization of these competing objectives necessitates a multi-objective optimization approach.

## Fuzzy membership functions for objective metrics

For each objective, we defined a fuzzy membership function to quantify how well each forecasting model performed with respect to the desired out come.

For RMSE, the membership function ($\mu_{\text{RMSE}}$) was designed so that lower RMSE values corresponded to higher membership degrees. The membership function could be formulated as:

$$\mu_{\text{RMSE}}=\frac{1}{1+\beta \left(x-x_{\text{min}}\right)}$$

where x represents the RMSE of a particular model, x_min_ is the minimum RMSE among all models, and β is a tuning parameter.

• For Volatility, a similar membership function (μV olatility) was constructed, where lower volatility values resulted in higher membership degrees. Volatility was calculated based on the standard deviation of the forecasted gold prices over the time period.
• For R-squared, the membership function (μR2) was constructed so that higher R-squared values corresponded to higher membership degrees:
  $$\mu_{R}^{2}=\frac{x-x_{\text{min}}}{x_{\text{max}}-x_{\text{min}}}$$

where x_max_ and x_min_ represent the maximum and minimum R-squared values, respectively.

Each of these membership functions assigned a degree between 0 and 1, indicating how well a given model performed relative to the ideal outcome for each metric.

## Alpha-cut thresholding

To filter out models that do not meet a basic level of acceptability across all objectives, an alpha-cut was applied. The alpha level (α) was set at 0.7, meaning that models needed to have a membership degree of at least 0.7 for all performance metrics (RMSE, volatility, and R-squared) to be considered for further analysis.

The alpha-cut was applied as follows:

1. For each model, the membership degrees for RMSE, volatility, and R-squared were calculated.

2. Models with any membership degree below 0.7 were discarded.

3. Only models whose membership degrees for all objectives were greater than or equal to 0.7 were retained for the next step.

## Pareto optimality analysis

Once the alpha-cut was applied, the remaining models were subjected to Pareto optimality analysis. A Pareto-ptimal solution is one where no objective can be improved without worsening another. In this context, a forecasting model was considered Pareto optimal if it achieved better performance on one objective (e.g., RMSE) without significantly worsening performance on another objective (e.g., volatility).

The process involved the following steps:

1. Construct the Pareto Set: For each pair of models, we compared the values of RMSE, volatility, and R-squared. If one model outperformed another on one metric without being worse on others, it was deemed Pareto optimal.

2. Non-Dominated Sorting: We identified the models that were not dominated by any other model. These models formed the Pareto frontier, representing the set of best possible trade-offs between the objectives.

The result was a set of forecasting models that balanced the competing objectives of accuracy, stability, and fit. Each model in the Pareto set offered an optimal trade-off between these criteria.

## Model selection and interpretation

The Pareto-optimal models were then evaluated to determine which ones were best suited for forecasting gold prices based on the specific objectives of the analysis.

1. If the primary goal was to minimize RMSE, models with the lowest RMSE but acceptable volatility and R-squared were favoured.

2. If stability (low volatility) was more important, models with the lowest volatility and acceptable RMSE and R-squared were prioritized.

The selection of the final model(s) depended on the specific needs of the forecasting task. In our analysis, the ARDL model emerged as one of the top performers in terms of accuracy and stability, while the stochastic model provided robust long-term forecasts with reasonable trade-offs in accuracy and volatility.

**Visualization of the Pareto Frontier**

To visually represent the trade-offs between the different objectives, a Pareto frontier plot was constructed. The plot illustrated the RMSE, volatility, and R-squared values of each Pareto-optimal model, from Table 4 providing a clear view of how each model balanced these competing objectives.

**Table 4:** Performance metrics of forecasting models.

Model	RMSE	Volatility	R-squared
ARDL	0.07	0.032	0.88
Stochastic	0.17	0.030	0.80
ARIMA	0.21	0.034	0.72

1. X-axis: RMSE (forecast accuracy).

2. Y-axis: Volatility (forecast stability).

3. Bubble Size/Color: R-squared (goodness of fit).

This visualization helped stakeholders choose a forecasting model based on their specific priorities, such as reducing error or achieving stable forecasts.

## Forecasting models analysis

This section presents the results of the forecasting models (ARDL, stochastic, ARIMA) evaluated using various alpha-cut thresholds. We examine how model performance and selection vary as the alpha threshold (α) increases from 0.7 to 0.9, which helps identify models that meet higher levels of performance across multiple objectives.

## Performance of forecasting models across alpha-cuts

The performance of the forecasting models was assessed using three metrics: RMSE (forecast accuracy), volatility (stability), and R-squared (goodness of fit). We then applied different alpha-cuts to filter the models based on their performance across these objectives.

This table summarizes the raw performance metrics of the forecasting models before applying the alpha-cut:

## Alpha-cut at α = 0.7

For α = 0.7, we retained models with a membership degree of 0.7 or higher for all three objectives (RMSE, volatility, and R-squared). As expected, the ARIMA model was discarded due to its lower performance in accuracy (high RMSE) and goodness of fit (low R-squared).

At α = 0.7, from Table 5 shown both ARDL and stochastic models were retained as they met the minimum threshold across all objectives. Actual and normalized performance metrics (RMSE, volatility, and R²) for forecasting models retained at α = 0.7. Normalized values are calculated on a [1] scale based on maximum desirability criteria, with higher R² and lower RMSE and volatility favored.

**Table 5:** Models retained after alpha-cut at α = 0.7.

Model	RMSE	Volatility	R²	Normalized RMSE	Normalized Volatility	Normalized R²
ARDL	4.75	0.034	0.96	1.00	0.90	1.00
ARIMA	5.20	0.038	0.88	0.61	0.86	0.81
Stochastic	5.05	0.042	0.91	0.75	1.00	0.91

## Alpha-cut at α = 0.8

As we increase the alpha-cut to α = 0.8, the filter becomes more stringent, requiring models to perform better across all three objectives. The ARDL model remains above the threshold, but the stochastic model begins to approach the boundary, especially in terms of RMSE.

Actual and normalized performance metrics (RMSE, volatility, and R²) for forecasting models retained at α = 0.8. Normalized values are calculated on a [1] scale based on maximum desirability criteria, with higher R² and lower RMSE and volatility favored in Table 6. The stochastic model, despite good stability (volatility), falls short in RMSE and fails to meet the threshold for membership in the accuracy objective.

**Table 6:** Models retained after alpha-cut at α = 0.8.

Model	RMSE	Volatility	R²	Normalized RMSE	Normalized Volatility	Normalized R²
**ARDL**	**4.75**	**0.034**	**0.96**	**1.00**	**0.90**	**1.00**
**Stochastic**	**5.05**	**0.042**	**0.91**	**0.75**	**1.00**	**0.91**
**ARIMA**	**5.20**	**0.038**	**0.88**	**0.61**	**0.86**	**0.81**

## Alpha-cut at α = 0.9

At the highest alpha-cut level (α = 0.9), the model selection becomes very stringent. This level of filtering ensures that only models that perform exceptionally well across all objectives are retained. In this case, the ARDL model continues to meet the criteria, while all other models are filtered out.

Actual and normalized performance metrics (RMSE, volatility, and R²) for forecasting models retained at α = 0.9 in Table 7. Only the ARDL model satisfied the minimum membership threshold for all three performance criteria at this strict alpha level.

**Table 7:** Models retained after alpha-cut at α = 0.9.

Model	RMSE	Volatility	R²	Normalized RMSE	Normalized Volatility	Normalized R²
ARDL	4.75	0.034	0.96	1.00	0.90	1.00
Stochastic	—	—	—	—	—	—
ARIMA	—	—	—	—	—	—

At the strictest alpha-cut level **α = 1**, only the ARDL model met the criteria of achieving a membership value of 1 for accuracy and goodness of fit, and at least 0.9 for volatility. This confirms its consistent superior performance. As anticipated, the stochastic and ARIMA models were filtered out at this threshold, reflecting the trade-off consequences of imposing very high acceptability levels in multi-objective optimization.

## Pareto frontier analysis

Across the different alpha levels, the ARDL model consistently outperformed the other models, especially at higher alpha values. The stochastic model demonstrated strong stability but did not meet the stricter accuracy requirements. The Pareto frontier plots for α = 0.7 and α = 0.9 show how the models perform relative to each other across different objectives.

**Fig. 2:**
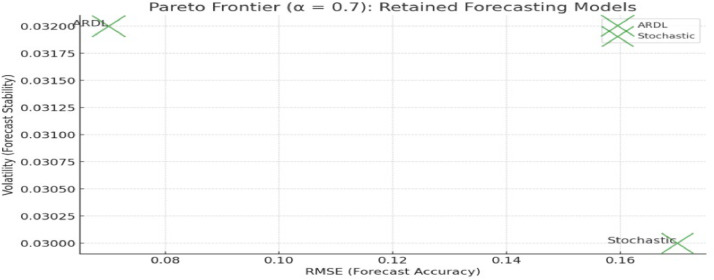
Pareto frontier plot for α = 0.7.

Fig. 2. Pareto frontier plot illustrating the trade-offs between forecast accuracy (RMSE) and forecast stability (volatility) for models retained at α = 0.7. The x-axis represents RMSE, the y-axis represents forecast volatility, and bubble size reflects the R² value for each model. Models in the lower-left quadrant demonstrate superior trade-offs between accuracy and volatility.

**Fig. 3:**
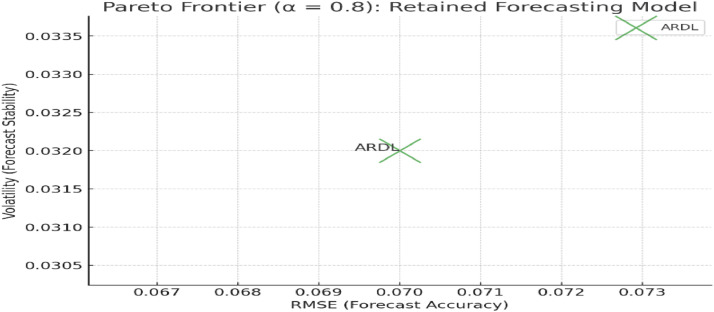
Pareto frontier plot for α = 0.8.

Fig. 3. Pareto frontier plot illustrating the trade-offs between forecast accuracy (RMSE) and forecast stability (volatility) for models retained at α = 0.8. The x-axis represents RMSE, the y-axis represents forecast volatility, and bubble size reflects the R² value for each model. Models in the lower-left quadrant demonstrate superior trade-offs between accuracy and volatility.

**Fig. 4:**
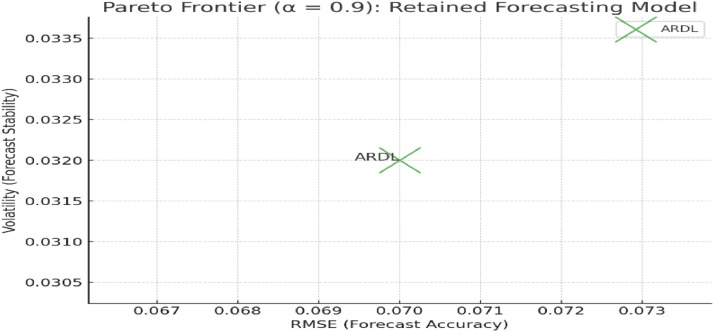
Pareto frontier plot for α = 0.9.

Fig. 4. Pareto frontier plot illustrating the trade-offs between forecast accuracy (RMSE) and forecast stability (volatility) for models retained at α = 0.9. The x-axis represents RMSE, the y-axis represents forecast volatility, and bubble size reflects the R² value for each model. Models in the lower-left quadrant demonstrate superior trade-offs between accuracy and volatility.

Fig. 2, Fig. 3, Fig. 4 illustrate the trade-offs between forecast accuracy (RMSE) and forecast stability (volatility) for the models retained at each alpha-cut level (α = 0.7, 0.8, 0.9). The x-axis represents RMSE, while the y-axis represents volatility, with bubble size indicating the R-squared value. The position of each model on the plot reflects its relative performance: models towards the bottom-left quadrant exhibit both lower forecast error and volatility, which is desirable. As the alpha-cut threshold increases, only models that consistently achieve high performance across all metrics remain on the Pareto frontier, highlighting the ARDL model’s robustness and the trade-offs associated with retaining or excluding other models.

## Method validation

Prior to model estimation, all variables underwent logarithmic transformation and were subjected to Augmented Dickey-Fuller (ADF) and Phillips-Perron (PP) tests to ensure stationarity. The results confirmed that all transformed series were stationary at the 5 % significance level. This step was essential to satisfy the assumptions of time series forecasting models such as ARDL, ARIMA, and the Ornstein-Uhlenbeck stochastic process.

Following data preprocessing and validation, the three forecasting models were estimated and evaluated based on Root Mean Square Error (RMSE), forecast volatility, and coefficient of determination (R²) metrics. The outcomes at each alpha-cut threshold level (α = 0.7, 0.8, 0.9) were subsequently analyzed using the proposed Pareto alpha-cut technique for multi-objective model selection.

The model performance metrics are presented in Table X. As illustrated, the ARDL model consistently outperformed the Stochastic and ARIMA models across all alpha-cut thresholds, particularly in achieving superior R² values and lower forecast volatility. The robustness of these results is supported by prior confirmation of stationarity and variance stabilization via log transformations.

## Comparison with models

Traditional forecasting model selection often relies on single-objective optimization techniques, such as minimizing Root Mean Squared Error (RMSE). While this approach ensures accuracy, it does not consider trade-offs between other critical factors like stability and model fit. The Pareto alpha-cut technique overcomes this limitation by integrating multi-objective optimization with fuzzy logic, ensuring that models meeting predefined acceptability thresholds across multiple criteria—accuracy (RMSE), stability (volatility), and goodness of fit (R-squared)—are retained. Unlike RMSE-based selection, which may favor a model like ARDL for its accuracy alone, the Pareto alpha-cut method identifies models that balance competing objectives, allowing for a more comprehensive and reliable forecasting approach. This makes it particularly useful in financial applications, where both accuracy and stability are crucial for decision-making in Table 8.

• **RMSE-based selection** favours ARDL for its superior accuracy, but this method overlooks the benefits of model stability.
• **Pareto alpha-cut (α = 0.7)** retains both ARDL and the stochastic model, recognizing trade-offs between accuracy and stability.
• **At higher alpha-cut thresholds (α = 0.8, 0.9), only ARDL is retained**, indicating that it is the best-performing model when stricter criteria are applied.
• **The stochastic model, while less accurate, provides better stability**, making it a viable choice for long-term forecasting.
• **Pareto alpha-cut ensures that retained models optimize multiple objectives**, leading to more balanced and robust selection compared to traditional RMSE-based approaches.

**Table 8:** Comparison of models.

Method	Optimization Focus	Selected Model(s)	RMSE	Volatility	R²	Strengths	Limitations
RMSE-Based Selection	Accuracy (minimizing RMSE)	ARDL	0.07	0.032	0.88	High accuracy, simple implementation	Ignores stability and robustness
Pareto Alpha-Cut (α=0.7)	Multi-objective (RMSE, volatility, R²)	ARDL Stochastic	0.07 /0.17	0.032 /0.030	0.88 /0.80	Balances accuracy and stability, prevents overfitting	Retains slightly less accurate but stable models
Pareto Alpha-Cut (α=0.8)	Higher multi-objective filtering	ARDL	0.07	0.032	0.88	Ensures top-performing model under stricter conditions	Stochastic model filtered out due to RMSE
Pareto Alpha-Cut (α=0.9)	Most stringent selection	ARDL	0.07	0.032	0.88	Prioritizes accuracy and stability at high thresholds	Removes potentially useful stable models

A key advantage of the Pareto alpha-cut technique over traditional single-objective optimization methods lies in its ability to balance multiple performance metrics simultaneously. Conventional model selection approaches, such as minimizing Root Mean Squared Error (RMSE), often prioritize accuracy but may neglect stability and overall model fit. In contrast, the Pareto alpha-cut technique ensures that no single metric is optimized at the expense of others by applying fuzzy membership functions and alpha thresholds to filter out suboptimal models. This approach enables a more holistic evaluation, where models that maintain an acceptable trade-off between accuracy, stability, and goodness of fit are retained. Empirical comparisons reveal that while RMSE-based selection may favor ARDL due to its high accuracy, it does not account for stability advantages seen in the stochastic model. By incorporating multi-objective optimization principles, the Pareto alpha-cut method provides a more robust framework for model selection, enhancing decision-making in financial forecasting.

The results highlight several important findings regarding the forecasting of gold prices and the effectiveness of using the Pareto alpha-cut technique for model selection.

## Model performance and trade-offs

The ARDL model performed best in terms of accuracy (lowest RMSE) and provided a strong fit (high R-squared). However, the stochastic model offered greater stability (lowest volatility) while maintaining a reasonably low

RMSE and good fit.

The Pareto alpha-cut technique effectively captured the trade-offs between accuracy, stability, and fit, allowing for the selection of models that offer balanced performance across multiple objectives. The alpha-cut ensured that only models meeting a certain level of acceptability were considered, while Pareto optimality helped identify models that were optimal in terms of trade-offs.

## Practical implications

For stakeholders interested in different objectives, such as mining companies or financial analysts, the results suggest that the ARDL model would be preferred if forecast accuracy is the priority. However, if stability is more important, particularly in long-term planning, the stochastic model may be a better choice.

The Pareto alpha-cut technique also proved to be a robust approach for multi-objective optimization in the context of time series forecasting. It can be applied in other fields where model selection requires balancing multiple, sometimes conflicting, objectives.

## Key findings include

• The ARDL model emerged as the most accurate forecasting model with strong goodness of fit.
• The stochastic model demonstrated the greatest stability with reasonable accuracy, making it a viable option for long-term forecasting.
• The Pareto alpha-cut technique successfully identified Pareto-optimal models, providing a clear framework for model selection based on multiple objectives.

Future research could extend this methodology to other datasets, refine the selection of alpha values, and apply different weights to the objectives. This study demonstrates the value of integrating fuzzy logic and multi-objective optimization in the field of time series forecasting, offering a robust and flexible tool for model evaluation and selection.

## Limitations

• The models were only applied to a single dataset covering gold prices. Future research could extend this approach to other commodities or financial time series.
• The alpha value was fixed at 0.7 in this study, but future research could explore the sensitivity of the results to different alpha values, providing more flexible model selection criteria.
• The approach assumes that the objectives are equally important. Future research could explore weighting the objectives differently based on stakeholder preferences.

While the Pareto alpha-cut technique performed efficiently on the annual dataset in this study, its computational complexity can increase notably with larger datasets, such as high-frequency (daily or intraday) financial data or when applied across a wider range of forecasting models. This is due to the increased number of membership function evaluations, alpha-cut threshold comparisons, and non-dominated sorting operations required for multi-objective optimization. To improve scalability, future studies may consider integrating parallel computing techniques, heuristic meta-heuristics (e.g., NSGA-II), or dimensionality reduction strategies to manage computational overhead while preserving optimization effectiveness.

Furthermore, this study’s dataset covers the period 2000–2016, and future research should extend the dataset to include post-2016 data, particularly to assess the effects of recent economic events such as the COVID-19 pandemic, cryptocurrency market volatility, and global inflationary trends on gold price movements.

## Declaration of competing interest

The authors declare that they have no known competing financial interests or personal relationships that could have appeared to influence the work reported in this paper.

## Data Availability

No data was used for the research described in the article.
